# *Lactobacillus*: Friend or Foe for Systemic Lupus Erythematosus?

**DOI:** 10.3389/fimmu.2022.883747

**Published:** 2022-05-23

**Authors:** Weijie Wang, Yongsheng Fan, Xinchang Wang

**Affiliations:** ^1^Department of Rheumatology, The Second Affiliated Hospital of Zhejiang Chinese Medical University, Hangzhou, China; ^2^School of Basic Medical Science, Zhejiang Chinese Medical University, Hangzhou, China

**Keywords:** *Lactobacillus*, systemic lupus erythematosus (SLE), immunoregulators, pathogenesis, microbiota

## Abstract

The cause of Systemic Lupus Erythematosus (SLE) remains largely unknown, despite the fact that it is well understood that a complex interaction between genes and environment is required for disease development. Microbiota serve as activators and are essential to immune homeostasis. *Lactobacillus* is thought to be an environmental agent affecting the development of SLE. However, beneficial therapeutic and anti-inflammatory effects of *Lactobacillus* on SLE were also explored. The discovery of *Lactobacillus* involvement in SLE will shed light on how SLE develops, as well as finding microbiota-targeted biomarkers and novel therapies. In this review, we attempt to describe the two sides of *Lactobacillus* in the occurrence, development, treatment and prognosis of SLE. We also discuss the effect of different strains *Lactobacillus* on immune cells, murine lupus, and patients. Finally, we try to illustrate the potential immunological mechanisms of *Lactobacillus* on SLE and provide evidence for further microbiota-targeted therapies.

## Introduction

Systemic lupus erythematosus (SLE) is an autoimmune, multisystem disorder that leads to tissue damage involving almost every organ (1). The estimated worldwide prevalence of SLE is about 30-50/100,000 (2, 3) and it is larger in developing countries (4). The 10-year survival rate is about 90%, seriously affecting the physical and mental health and quality of life of patients (5). The adaptive immune system is dysregulated in SLE, resulting in a variety of abnormalities including as autoantibody production, activation of auto aggressive T effector cells and B cells, and a reduction in regulatory T cells (Tregs). SLE treatment includes glucocorticoids (GCs), antimalarial medicines, nonsteroidal anti-inflammatory drugs (NSAIDs), immunosuppressive agents, and B cell–targeting biologics and Chinese herbal medicine (6). However, the side effects such as gastrointestinal reaction, metabolic disorders and infections enable the doctors and scientists to seek new therapies. Recently, there were a large number of evidence about the relationship between the gut microbiota especially *Lactobacillus* spp. and SLE (7–9).

SLE is distinguished by the presence of high-titer serological autoantibodies, such as antibodies that bind to double-stranded DNA (dsDNA), SmRNP, SSA/Ro, and SSB/La (10). Bagavant et al. found that *Aggregatibacter actinomycetemcomitans, Porphyromonas gingivalis*, *Treponema denticola*, and the *commensal Capnocytophaga ochracea* have higher expression in SLE patients with higher anti-dsDNA antibody levels compared to patients without anti-dsDNA. Furthermore, Anti-SmRNP antibodies were shown to be related with increased anti-bacterial antibody titers against all the bacteria. However, anti-SSA/Ro and anti-SSB/La failed to associate with any of the periodontal bacteria (11).

The *Lactobacilli*, which are used commercially in the production of fermented foods, are an important component of the microbiota of human beings. Recently, *Lactobacilli* is thought to be an environmental agent affecting the development of SLE (12, 13). It was found that the *Lactobacillus* were significantly enriched in active SLE patients compared with healthy controls and rheumatoid arthritis (RA) patients in China (14, 15). Moreover, *Lactobacillus reuteri* exacerbated lupus-related pathogenesis in TLR7-dependent Mice (16). Particularly, women of reproductive age are nine times more likely than males to be diagnosed with SLE (10). Given that there is a link between sex and disease, gaining a better understanding of gender differences in gut microbiota will aid in interpreting therapeutic options. In MRL/lpr mice, Zhang H, et al. found females had significantly higher *Lactobacillaceae* and *Streptococcaceae* levels than did male mice. Importantly, retinoic acid improved lupus symptoms in MRL/lpr mice by restoring *Lactobacillus* spp. gut colonization (17).

There were also some recent studies on the oral microbiota of SLE. A preliminary study of Chinese SLE patients found that family *Lactobacillaceae* and the genus *Lactobacillus* were increased in patients with SLE (18). *Actinomyces* and *Lactobacillus* relative abundance in oral samples was substantially linked with their relative abundance in fecal samples. The difference in *Lactobacillus* relative abundance between SLE and healthy controls was significant in oral samples but not in fecal samples (19).

However, the variable effects of *Lactobacilli* on SLE highlight their dual role as beneficial or harmful depending on the context (20). In newly diagnosed SLE Egyptian patients, *Lactobacillus* showed a significant decrease (7). Several species of *Lactobacilli* can confer a range of beneficial effects on the host of SLE. For example, *L. delbrueckii* and *L. rhamnosus* have been shown to be effective in the elevation of Tregs and the decrease of inflammatory cytokines and disease severity in SLE‐induced mice (21). Mu et al. found that *Lactobacillus* spp. supplementation plays an anti-inflammatory role through reducing IL-6 and enhancing IL-10 generation in the intestine (22).

In order to understand the different roles of species of *Lactobacilli* and how the proper species of *Lactobacilli* can be targeted as therapy for SLE, we review prior previous findings regarding *Lactobacillus* in SLE patients and murine lupus models as well as the immune cells relevant to SLE. In addition, we will highlight the possible approaches to investigate the different species of *Lactobacilli* and to find the new way for microbiome-based precision medicine.

## The Potential Immunological Mechanisms of Lactobacillus on SLE

Probiotics, including *Lactobacillus* species, favorably impact the host organism by enhancing and stabilizing gut flora (23). *Lactobacillus* might cling to intestinal epithelial cells and produce antibiotic chemicals, inhibiting the growth of dangerous germs. They can boost the nonspecific cellular immune response by activating macrophages, natural killer cells, and releasing numerous cytokines, as mentioned in the immune cells part. Moreover, they can boost the immune system of the gut mucosa by raising the number of IgA^+^ cells (24).

*Lactobacillus* adheres to the gastrointestinal mucosa by means of an interaction with toll-like receptors (25), by surface proteins and fatty acids (*L. rhamnosus* PEN) (26), and by proteinaceous surface layer components (*L. plantarum* 91) (27) to trigger the host’s immune response. Furthermore, they could upregulate tight-junction (TJ) proteins and MUC2, MUC3 and MUC1 in colonic epithelial cells and elevate butyrate levels, as well as microbiome modulation (28).

*Lactobacilli* can produce cytoprotective compounds, including short chain fatty acids, (SCFAs). SCFAs which act as local energy carriers for bacteria and gut epithelial cells, have emerged as key mediators in in the relationship between diet, gut microbiota, and human health (29, 30). SFCAs could stimulate the generation of retinoic acid by epithelial cells (31), a vitamin A-derived metabolite that cooperates with TGF-β to enhance Treg differentiation (32) and prevent Th17 cell differentiation (33). Studies have revealed several mechanisms through which retinoic acid treatment may modulate the course of SLE. For example, retinoic acid has been shown to restore the *Lactobacilli* in MRL/lpr mice and ameliorate the inflammatory symptoms of lupus (34, 35). In addition, retinoic acid suppresses the signaling of transcriptional regulators needed for lupus onset. For example, retinoic acid could inhibit prolyl isomerase Pin1, a regulator of IL-1 receptor-associated kinase 1 (IRAK1) in the TLR7/TLR9 signaling pathway. Moreover, interferon regulatory factor 7 (IRF-7) signaling, which has been linked to lupus progression, was also reported inhibited by retinoic acid (36).

**Figure 1:**
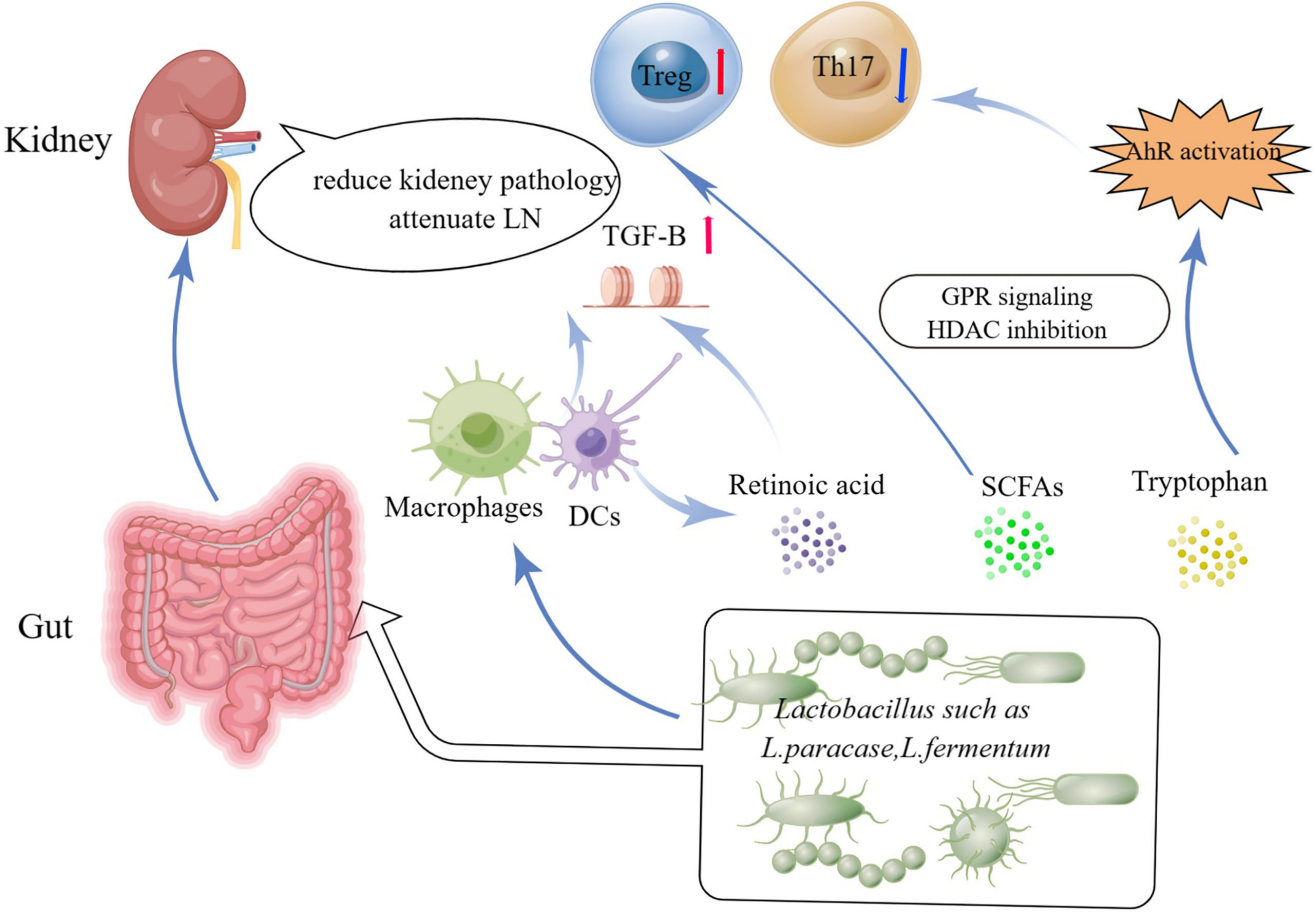
Potential beneficial functions of *Lactobacillus* and *Lactobacillus*-derived bioactive metabolites in the treatment of SLE and the communication pathways on *Lactobacilli*-kidney axis. HDACs, histone deacetylases; GPCRs, ligands for G-protein-coupled receptors; SCFAs, Short-chain fatty acids.

Tryptophan is an important amino acid that some *Lactobacilli* strains convert into strong immune-modulating compounds that bind the Aryl hydrocarbon receptor (AhR). Tryptophanase-positive bacteria, such as *Lactobacillus reuteri*, produce various indole metabolites from dietary tryptophan that can stimulate AhR activity (37). AhR is a transcription factor found in the cytosol of a variety of cells. It has been observed that activating AhR regulates the methylation status of FoxP3 and IL-17 gene promoters and ameliorated experimental colitis (38). Recent research found considerable AhR activation in the peripheral blood of active SLE patients (39) (**Figure 1**).

## From Gut-Kidney Axis to *Lactobacilli-*Kidney *axis*

SLE is a chronic autoimmune disease that affects the kidneys in approximately half of all patients. Lupus nephritis (LN) is a significant risk factor for SLE morbidity and death (40). LN were linked to intestinal wall congestion, metabolic acidosis, frequent antibiotic usage, all of which have an influence on intestinal tight junctions and led to an increase in bacterial metabolic products crossing the intestinal barrier (41, 42). Moreover, the increased gastrointestinal urea output led in gut microbial dysbiosis. Furthermore, urea supplementation in drinking water related to changes in gut flora (43). Mu et al. and his colleagues demonstrated that a mixture of 5 *Lactobacillus strains* (*Lactobacillus oris, Lactobacillus rhamnosus, Lactobacillus reuteri, Lactobacillus johnsonii, and Lactobacillus gasseri*) restored the mucosal barrier function and reduced kidney pathology by increasing Treg cells and suppressing pathogenic Th17 cells (22). Future studies about *Lactobacillus* treatment in SLE patients are warranted to determine and needed (**Figure 1**).

## *Lactobacillus* Are Altered in SLE Patients

Although there were few randomized controlled trials (RCTs) about the *Lactobacillus*’s effect on SLE patients, a large amount of cohort clinical studies on the relation between *Lactobacillus* and SLE were increasing. Newly diagnosed treatment-naive SLE cases (44) and SLE patients that had not received glucocorticoid (GC) therapy (45) possessed a decrease of *Lactobacillus* in feces in Chinese and Egyptian patients compared with the healthy controls. However, SLE patients in most cohorts both active and remissive, both adults and children all manifested the enrichment of *Lactobacillus* in both fecal and oral sample in China and Netherlands (14, 15, 18, 19, 46) (**Table 1**). Notably, *Lactobacillus* enriched in active SLE patients and lupus erythematosus skin lesions patients (47) as disease severity might play a role in abundance (14). It is hard to illustrate whether the *Lactobacillus* correlated with disease severity or higher dose of GC treatment. Moreover, SLE patients received GC therapy also have abundance in *Lactobacillus* (45). To illustrate the problem, more RCTs about the Lactobacillus’s effect on SLE patients should be recommended.

**Table 1:** The cohort studies on Lactobacillus in SLE patients.

Study	Location	Inclusion criteria	SLE/HC (patients)	Microbiota (species of *Lactobacillus*)	correlation between SLEDAI and *Lactobacillus* abundance
Yao Li, et al., 2019 (15)	China	19 active patients (SLEDAI>8), 21 remissive patients	40 SLE/20RA/22HC	Fecal samples: enrichment of *Lactobacillus*;	*Lactobacillus* enriched in active SLE patients
Mengchen Guo, et al., 2020 (45)	China	20SLE received GC;17 SLE non-GC	20SLE+G/17SLE-G/20HC	Fecal samples: *Lactococcus* Decrease in SLE-G, enriched in SLE+G	Associated with glucocorticoid therapy
Bao-Zhu Li, et al., 2020 (18)	China	20 SLE patients	20/19	Oral samples: *Lactobacillus* increased	NA
Ying Zhao, et al., 2020 (47)	China	20 lupus erythematosus dermatology patients	20/20	Fecal samples: enrichment of *Lactobacillus*	NA
Fengping Liu, et al.2021 (14)	China	active and remissive SLE; and patients currently receiving low-dose prednisone (maximum: 7.5 mg daily) and hydroxychloroquine.	35/35	Fecal samples: enrichment of *Lactobacillus*;*L. iners* was elevated.	disease severity might play a role in abundance of *Lactobacillus*
Marian A. Gerges, et al., 2021 (44)	Egypt	Newly diagnosed, treatment-naive SLE cases	20/20	Fecal samples: Decrease of *Lactobacillus*;	an inverse but nonsignificant correlation
Taco A van der Meulen, et al. 2021 (19).	Netherlands	30SLE	30SLE/39PSS/965HC	Fecal and oral samples: *Lactobacillus* enriched in SLE and PSS	NA
Min Wen, et al., 2021 (46)	China	Children with SLE/LN	33/28	Fecal samples: enrichment of *Lactobacillales*	NA

SLE, Systemic Lupus Erythematosus; RA, Rheumatoid Arthritis; PSS, primary Sjögren’s syndrome; HC, health controls; LN, lupus nephritis; GC, glucocorticoids; NA, none applicable.

## The Role of *Lactobacillus* on Immune Cells

### Macrophages Especially Kidney Macrophages

In SLE, IgG antibodies induce inflammation and organ damage. Recent research suggests that inhibiting FcR-dependent macrophage activation may be an effective therapy strategy for SLE (48, 49). Inhibiting kidney macrophage glycolysis reduced autoantibody-induced inflammation, which may have therapeutic implications for lupus nephritis(LN) (50). Four Probiotic *Lactobacillus* strains (*Lactobacillus rhamnosus GG, L. rhamnosus KLSD, L. helveticus IMAU70129, and L. casei IMAU60214*) induced early proinflammatory cytokines such as IL-8, TNF-α, IL-12p70, and IL-6 and activated human macrophages. Moreover, the NF-κB pp65 and TLR2-dependent signaling were also increased by treatment with the strains (51). In addition, *Lactobacillus salivarius* strains induced cytokine responses that are Toll-like receptor2 (TLR2) independent but myeloid differentiation primary response88 (MyD88) dependent in both human and mouse macrophages with the upregulation of Tlr1, Tlr2 and Clec4e PRR genes (52). TLR-MyD88 pathway has been proved to be involved in the pathogenesis of SLE (53, 54). Therefore, some species of *Lactobacillus* may be beneficial for SLE through the pathway of TLR-MyD88 targeting on the kidney macrophages.

### Dendritic Cells

SLE is associated with excessive uncontrolled inflammatory immunological responses that result in immune tolerance loss. Dendritic cells (DCs) are critical immune cells that govern immune responses. Renal DCs and macrophages contribute to chronic inflammation in LN. The involvement of these cells in SLE kidneys sparked interest when studies revealed that interstitial CD68^+^ mononuclear phagocyte infiltration is related with a worse prognosis in patients (55, 56). Several subtypes of these cells (CD103^+^ DCs, myeloid DCs, inflammatory Ly6Chi macrophages, and resident F4/80hi/Ly6Clo macrophages) have been identified by immunohistochemistry in LN biopsies (57) and by flow cytometry in mouse SLE models (58). In recent research, after treatment with GM-CSF and IL-4, together with *L. delbrueckii and L. rhamnosus*, the expression of indoleamine 2,3dioxygenase (IDO) and IL-10 increased, but IL-12 dropped. IDO was substantially expressed in mature DCs from SLE patients (59). Moreover, *L. delbrueckii* and *L. rhamnosus* could decrease expression of CXCR3, CCR5, CCR4, and CCR3 on the tolerogenic phenotype of DCs in SLE patients (60). Another study showed that *Lactobacillus plantarum NCU116* increased the expression levels of Th17, Treg and specific transcription factors RORγt and Foxp3 in CTX-induced immunosuppression mice. In addition, it boosted the number of CD4^+^T cells and DCs considerably and enhanced the expression of Toll-like receptors (TLRs) (61).

### B Cells

SLE is associated with B cell hyperactivity (62). BAFF (the B cell activating factor) over-expression has been commonly detected among patients with SLE and seems to be strongly involved in the pathogenesis of SLE (63). Belimumab (Benlysta^®^), which is the only biological agent currently approved for the treatment of SLE, has been used worldwide (64).The systemic review provided the evidence for the efficacy and good response of Belimumab. However, the adverse effects could not be reduced compared with the standard care (65). The findings on the effect of *Lactobacillus* to B cells could shed some light on reducing the gastrointestinal side effects when added into treat SLE. Studies showed that *Lactobacillus rhamnosus* (LGG) intervention can promote the development and function of B lymphocytes by increasing the expression of CD40, CD80 and MHC-II on B cell surface and activating the secretion of IgA and IgM as well as decreasing the IgG (66). In addition, a randomized trial demonstrated that LGG has an immediate effect in the human gut with upregulating genes related to B cell activation (67). Follicular helper T (Tfh) cells, which participate in the transmission of information in the process of B cell differentiation, assist in the activation of B cells. After administration of *Lactobacillus paracasei* MCC1849, IgA secretion was elevated and Follicular helper T (Tfh) cells was induced in the BALB/c mice. Moreover, MCC1849 boosted the expression of genes involved in Tfh cell development, such as IL-12p40, IL-10, IL-21, STAT4, and Bcl-6 (68).

### T Cells

With age, B220^+^CD4^−^CD8^−^ double-negative (DN) T cells increase in peripheral lymphoid organs of MRL/lpr mice. *Lactobacillus casei* was found to lower B220^+^ T cells to prevent the lymphadenopathy and prolong the lifespan of MRL/lpr mice (69). Regulatory T cells (Tregs) and Helper T cells (Ths) play a significant role in the control of immune responses and induction of peripheral tolerance. Dysregulation of Tregs and Th1/Th2/Th17 are involved in the pathogenesis of SLE. Many species of *Lactobacillus* have been reported to reprogram the CD4^+^ T cells into immunoregulatory T cells as well as regulating the Th cells and regulatory T cells. Marco Colonna and his colleagues showed that the *Lactobacillus reuteri* provides indole-3-lactic acid, that activate AhR and lead to inducing immunoregulatory T cells -gut intraepithelial CD4^+^CD8αα^+^ T cells (70). When pristane-induced lupus mice were administrated *L. delbrueckii and L. rhamnosus*,the disease severity was ameliorated by upregulating the Treg cells and decreasing anti-dsDNA and IL-6 (21). In addition, *Lactobacillus sakei* WIKIM30 was reported to ameliorate atopic dermatitis by decreasing the levels of Th2 cytokines (IL-4, IL-5, and IL-13) and enhancing Treg production and increasing the relative abundance of Treg-promoting bacteria (71). Moreover, *Lactobacillus rhamnosus GG* relieve allergies in mice by regulating the balance of T/B cells and Th1/Th2 and Th17/Treg cytokines (72). When *Lactobacillus acidipiscis* was orally administrated in a mouse model of experimental autoimmune encephalomyelitis (EAE), the generation of regulatory γδ T cells was induced and Th1 and Th17 cells decreased (73). Lactobacillus has been proved to modulate the Th17 cells in ameliorating many autoimmune and metabolic diseases such as SLE, hypertension, EAE, rheumatoid arthritis and colitis (74–78).

## The Role of *Lactobacillus* on Animal Models of SLE

There was still a controversial issue on whether the changes in the microbiome communities in Lupus are direct drivers of pathogenesis. Interestingly, transplanting fecal samples from mice with a Lupus-like syndrome into germ-free mice induced higher increased levels of anti-dsDNA antibodies and encouraged an inflammatory alteration in recipient mice’s immune systems (79). Early and short-term antibiotics exposure exacerbated lupus severity by diminishing beneficial gut microbiota for lupus including *Lactobacillus (* 80).

Mouse models of SLE can be divided into four groups: spontaneous models, induced models, genetically modified models, and humanized mouse models (81). Spontaneous mice models, which have a good genetic background and genetic stability, mainly include NZB/WF1 model, MRL model, BXSB model, Palmerston North mice, NZB/KN model, NZM model, BXD2 model. Among which, the NZB/WF1 model and MRL model were the most classic ones and commonly used in the research (82).

**Figure 2:**
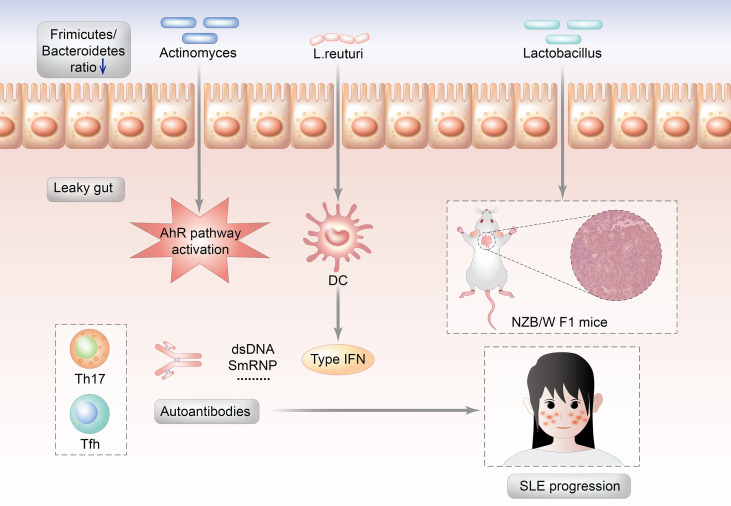
Potential mechanisms by which the Lactobacillus triggers the autoimmunity of SLE.

*Lactobacillus* abundance varies depending on the SLE animal models (MRL/lpr mice or NZB/WF1 mice). This might imply that interactions between gut microorganisms and the host, rather than the enrichment of specific gut microorganisms, are more important in the development of SLE. The gut microbiota of MRL/lpr lupus-prone mice are enriched in *Lachnospiraceae* and depleted in *Lactobacillaceae* (17). In MRL/lpr mice, *Lactobacillus* might play a preventive role in terms of lupus pathogenesis due to the relative absence before SLE onset. Moreover, *Lactobacillus* addition reduce proteinuria and autoantibodies and improve the renal condition in MRL/lpr mice (22). However, *Lactobacillus* play a contradictory role in NZB/WF1 mice. Luo et al. and his colleague investigated that the gut microbiota changed throughout the mouse lifetime in NZB/WF1 mice. The alterations were most noticeable before and after lupus onset. The relative abundance of *Lactobacillus* increased substantially during lupus development, which was reduced by dexamethasone. The relative abundance of *Lactobacillus* species was shown to be positively related to poorer renal function and higher-level systemic autoimmunity (83) (**Figure 2**).

Induced models of lupus include pristane induced lupus model, graft versus host disease induced lupus model, common human anti-DNA idiotype (16/6 id) induced lupus model, imiquimod/resiquimod induced lupus model, gut bacteria induced lupus model, anti-autoantibodies induced lupus glomerulonephritis model and environmental exposures induced lupus model. For example, resiquimod, Toll-like receptor 7/8 agonist, induced lupus model which were TLR7/9- dependent mice lupus models (82). *Lactobacillus. reuteri* increased over time in the feces of mice from both TLR7-dependent mice lupus models (16) as their disease progress and in the SLE patients (12) Moreover, *L. reuteri* worsens autoimmune manifestations by engaging type I interferon pathways in TLR7-dependent lupus mice. However, *Lactobacillus fermentum CECT5716* was reported to prevent hypertension and endothelial dysfunction in lupus model mice induced by imiquimod, the agonist of TLR-7 (84).

On the other hand, the beneficial anti-inflammatory and immunoregulation effect of different strains of *Lactobacilli* were explored in NZB/W F1 mice (85–89) and MRL/lpr mice (22). Some of the studies focused on the therapeutic effect of *Lactobacilli* on organ damage such as on cardiac tissue (85, 87), liver (86) and the kidney (22). Moreover, enhancement of Tregs and the decrease of Th17 and Th1 cells were explored as the immunoregulation effect of *Lactobacilli* in pristane-induced mice, MRL/lpr mice, and NZB/W F1 mice models (21, 22, 88, 90) (**Table 2**).

**Table 2:** Studies investigating the *Lactobacillus* in murine lupus.

Study	Animal model	Lactobacillus strain	Effect on immuneresponse	Potential cytokines and signals
Daniel F. Zegarra-Ruiz, et al., 2019 (16)	TLR7-dependent Mice	*Lactobacillus reuteri*	Exacerbated lupus-related pathogenesis	TLR7/IFN-pathway
Néstor de la Visitación, et al.2021 (84)	Lupus model induced by TLR7 activation	*Lactobacillus fermentum* CECT5716	Prevented the development of hypertension and endothelial dysfunction	Restored Th17/Treg balance in vascular tissues
Wei-Syun Hu, et al., 2017 (85)	NZB/W F1 mice	*Lactobacillus paracasei GMNL-32*	anti-fibrosis and anti-apoptotic effects on the cardiac tissue	Increased PI3K, Bcl-xl, and Bcl2 and reduce MMP-9 and COX2
Tsai-Ching Hsu, et al., 2017 (86)	NZB/W F1 mice	*Lactobacillus paracasei GMNL-32, Lactobacillus reuteri GMNL-89 and L.reuteri GMNL-263*	Ameliorated hepatic apoptosis and inflammatory indicators; Antioxidant activities	Reduced IL-1β, IL-6 and TNF-α in the livers MAPK-NFkB; Decreased TLR-4, TLR-5, TLR-7, TLR-9, MyD88, PI3K and AKT
Fatemeh Mardani, et al., 2018 (90) Sahar Khorasani, et al.2019 (21)	pristane‐induced mice	*L. delbrueckii (DEL) subsp. lactis (PTCC: 1743) and L. rhamnosus (RAM; ATCC: 9595)*	Delayed and controlled SLE	Decreased Th1 and Th17; enhancement of Tregs
Toral M, et al., 2019 (87)	NZB/W F1 mice	*Lactobacillus fermentum CECT5716*	Prevented endothelial dysfunction and vascular oxidative stress	Reduced B and T lymphocytes, IL-6, IL-1b, and TNF-a,
Bor-Show Tzang, et al., 2017 (88)	NZB/W F1 mice	*Lactobacillus reuteri GMNL-263 (GMNL-263)*	Antioxidant activities	Increased CD4^+^CD25^+^FoxP3^+^T cells.
Yeh YL, et al., 2021 (89)	NZB/W F1 Mice	*L. reuteri GMNL-263*	cardiac protective effects; inhibiting cardiac apoptosis and fibrosis.	PI3K/Akt pathway
A Mike, et al., 1999 (69)	MRL/lpr mice	*Lactobacillus casei*	Prevented the expansion of B220^+^ T cells	Reduced IL-6
Qinghui Mu, et al., 2017 (22)	MRL/lpr mice	a mixture of 5 *Lactobacillus* *strains (Lactobacillus oris, Lactobacillus rhamnosus, Lactobacillus reuteri, Lactobacillus johnsonii, and Lactobacillus* *gasseri)*	Anti-inflammatory Attenuated LN	Increased IL-10 and decreased IgG2a t Treg-Th17 balance increase of Muc2, ZO-1.
Da Som Kim, et al., 2021 (74)	MRL/lpr mice	*Lactobacillus acidophilus*	Decreased the proportion of double negative T Cells and renal inflammation	Modulated Th17/Treg balance *via* the SIGNR3 Pathway

SLE, Systemic Lupus Erythematosus; LN, lupus nephritis.

Until now, there were few studies on the roles of *Lactobacilli* on genetically modified models, and humanized mouse models of lupus. In the future, more microbiota research especially about *Lactobacilli* are needed in these two models, which imitate the cross- interaction and immunological damage more similar to SLE patients.

## Perspectives on *Lactobacillus* Treatment in SLE

### Putting FMT Into Clinical Trials on SLE Would Shed Light on Its Beneficial Effects

With respect to the data on *Lactobacillus*, it is still a controversial issue on whether they would be potential immunoregulators in SLE. Although fecal microbiota transplant (FMT) has been used in several lupus models, TLR-7 dependent lupus model and MRL/lpr mice, and achieved satisfactory results resulting to restore the gut microbiota and ameliorate the lupus severity (80, 91). It is still a long way to put FMT into clinical trials on SLE owing to the ethic and patient’s wishes. Many probiotics researches are supported by commercial probiotic business groups. Lack of sufficient medical evidence and the commercial interests make objective interpretation of the *Lactobacillus* as probiotics close to impossible. However, recent microbiome findings, as well as the advent of innovative high-throughput sequencing and testing approaches, may allow us to concentrate on patient-oriented therapies.

### *Lactobacillus*-Based Precision Medicine and Lactobacillus-Oriented Components in Food and Herbs May Have Great Potential to SLE Treatment

As glucocorticoids were still the most effective immunosuppressant and widely used for the treatment of SLE, *Lactobacillus*-based precision medicine would be a better adjuvant therapy to reduce the side effects. Zhixing He and his colleagues had proved that both the FMT of prednisone-treated MRL/lpr mice (92) and bromofuranone, inhibitor of AI-2 quorum sensing (93), could promote efficacy of prednisone and reduce the side effects through increasing the beneficial bacteria including *Lactobacillus*. Interestingly, orange intake was directly associated with *Lactobacillus* in non-active (SLEDAI score ≤ 8) Caucasian SLE patients (94). Hence, the low-fat/high-fiber diet and even the useful components *Lactobacillus-*oriented extracted from the orange or Chinese medicinal herbs along with the FMT would shed light on the treatment of SLE.

### Comparative Proteomics or Transcriptomics Are Encouraged to Explore the Two Sides of the *Lactobacillus*

Different strains of *Lactobacillus* pose different effects even completely opposite. Interaction between different strains to produce an effect would also be another focusing point. Comparative proteomics or transcriptomics on the exact difference about the structure of the strain may provide new evidence on the treatment of SLE. To our best knowledge, although there were no adverse effects on the RCT trials of *Lactobacillus* on inflammatory bowel disease (95) and rheumatoid arthritis (96), the adverse effects of *Lactobacillus* as probiotics must be further investigated utilizing additional antibiotic regimens, probiotic strain combinations, and human microbiome transplants into germ-free mice. Therefore, the potential long-term clinical consequences of *Lactobacillus*-induced dysbiosis could be assessed.

## Conclusions

This review shows that some beneficial strains of *Lactobacillus* have anti-inflammatory and immunoregulation effects on SLE and may become a better adjuvant therapy to reduce the side effects of the glucocorticoids and immunosuppressant. However, there are still many data on the harmful strains of *Lactobacillus* that contradicts this tendency. Since *Lactobacillus* could interact with the occurrence, development and treatment of SLE and different animal models of lupus, the question of whether *Lactobacillus* will be friend or foe could depend on the strain itself and the circumstances. Finally, we believe that we would witness useful medications and therapies about *Lactobacillus* on SLE in the future, when large cohort are available, and further studies might need to form national or even international randomized and blinded clinical trials.

## Author Contributions

WW contributed to the conception of the study and wrote the first draft of the manuscript. XW and YF revised the manuscript. YF supervised the review. All authors contributed to manuscript revision, read and approved the submitted version.

## Funding

This research was supported by National Natural Science Foundation of China (NO.82004238); Natural Science Foundation of Zhejiang Province (NO. LQ20H270007; LBY21H270001) and Zhejiang Medicine and Health Science and Technology Project (NO. 2021KY843).

## Conflict of Interest

The authors declare that the research was conducted in the absence of any commercial or financial relationships that could be construed as a potential conflict of interest.

## Publisher’s Note

All claims expressed in this article are solely those of the authors and do not necessarily represent those of their affiliated organizations, or those of the publisher, the editors and the reviewers. Any product that may be evaluated in this article, or claim that may be made by its manufacturer, is not guaranteed or endorsed by the publisher.
